# A L833V/H835L *EGFR* variant lung adenocarcinoma with skin metastasis: A case report and literature review

**DOI:** 10.1016/j.heliyon.2022.e12080

**Published:** 2022-12-05

**Authors:** Xian Yang, Yang Yao, Qing Zhu

**Affiliations:** a Department of Abdominal Oncology, Cancer Center, West China Hospital, Sichuan University, Chengdu 610041, China; b Department of Oncology, Suining City Central Hospital, Suining 610041, China

**Keywords:** Non-small cell lung cancer, Epidermal growth factor receptor, Compound variants, L833V/H835L, Gefitinib

## Abstract

The co-occurrence of two or more rare variants, known as compound variants, is rare in non-small cell lung carcinoma with epidermal growth factor receptor (*EGFR*) variants, and the compound variant L833V/H835L in exon 21 of EGFR is extremely rare. There is very little evidence regarding its treatment. Herein, we report a case of an advanced lung adenocarcinoma patient with cutaneous metastases. Next generation sequencing detected a combination variant of *EGFR* exon 21 L833V/H835L. To our surprise, our patient had almost complete remission of skin symptoms after 1 month of oral gefitinib (250 mg/d qd) treatment with less skin toxicity. At the time of this report submission, the last CT scan confirmed that the patient had achieved partial response. To date, the patient has achieved a remarkable result with a progression-free survival of 18 + months. The presentation of this case and a literature review suggest that tailored therapeutic interventions are available for this subset of patients.

## Highlights

• NSCLC with *EGFR* exon 21 L833V/H835L compound mutations are rare.
• Gefitinib was effective against L833V/H835L compound mutation.
• Tailored therapeutic interventions for rare compound variants may be an option.
• Establishment of a database of rare variants is necessary for patient management.

## 1 Introduction

Lung cancer is one of the most common malignancies and the leading cause of cancer-related death. With the application of epidermal growth factor receptor-tyrosine kinase inhibitors (EGFR-TKI) in non-small cell lung carcinoma (NSCLC), the prognosis of patients with EGFR variants has been significantly improved. However, the current information about EGFR-TKI treatment mainly focuses on classical variants, that is, exon 19 deletion variant and exon 21 L858R point variant, which account for 85% of the EGFR gene changes and have been confirmed to show good clinical response to EGFR-TKIs [1]. Relatively less information is available for rare variants, a heterogeneous group of genomic alterations [2]. With the popularity of next generation sequencing (NGS), more and more rare variants are being detected. Rare variants can be further divided into relatively rare variants, such as G719X, L861Q and S768I, also known as major rare variants, and extremely rare variants, such as L833V/H835L and other compound variants [3]. In some limited prospective studies (e.g., LUX-Lung 2, 3, and 6), most retrospective series, and case reports, afatinib has shown good efficacy in patients with major rare variants. However, compound variants are rare, and there are only a few small-sample retrospective studies or case reports; hence, it is almost impossible to carry out relevant clinical studies. Therefore, there is no unified treatment strategy for patients carrying these variants [4]. Herein, we describe an unusual case of L833V/H835L compound variant lung adenocarcinoma that metastasized to the skin at the time of initial diagnosis, which had clinical response to first-line gefitinib therapy. At present, progress-free survival (PFS) is more than 18 months after targeted therapy.

## 2 Clinical case

The patient was a 65-year-old male with a history of heavy smoking (≥40 cigarettes/day, ≥30 years) and alcohol consumption (≥100 g/day, ≥30 years). In July 2020, the patient developed itching and redness on the right side of the neck and shoulder after farming, which was treated as "allergic dermatitis" at a local clinic. However, the symptoms did not alleviate and he gradually developed skin redness, swelling, and hard lumps. He consulted a doctor at the Department of Dermatology in Suining City Central Hospital on September 16, 2020.

**Figure 1:**
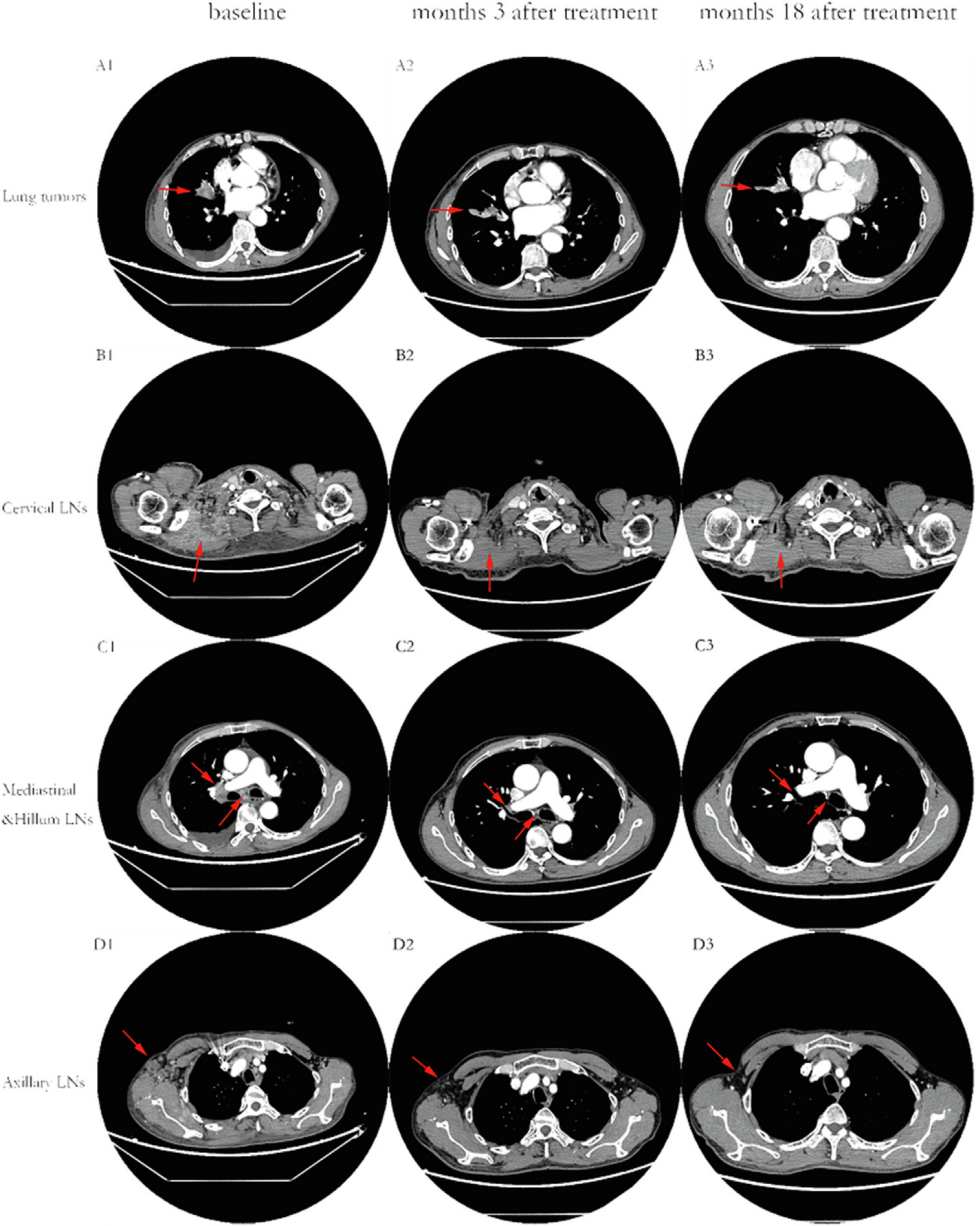
Partial response of CT image findings. (A) The primary foci in the right lung (red arrows) shrank significantly after three months of treatment. (B) The right cervical root lymph nodes (red circles) continued to shrink. (C) The edges of the enlarged lymph nodes in the right pulmonary hilar (large red circles) and mediastinum (small red circles) gradually became clear. (D) The right axillary lymph nodes (red circles) gradually became clearly demarcated from the surrounding tissue. Columns 1, 2, and 3 represent the CT image findings before the start of treatment, three months after treatment, and at the latest follow-up (18 + months), respectively.

**Figure 2:**
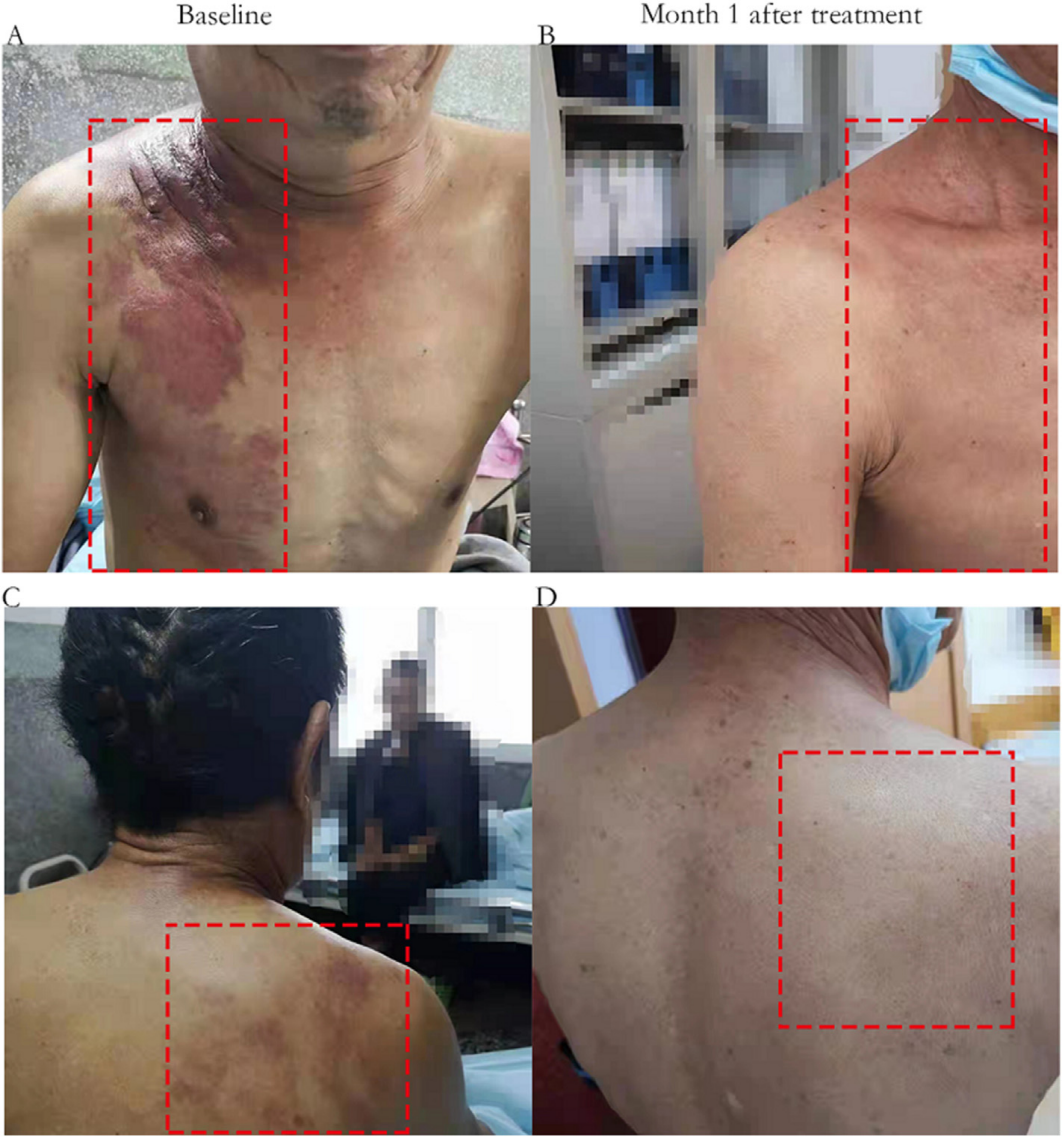
Changes in skin lesions before and after treatment. (A, B) Erythematous lesions on the anterior chest skin and shoulders disappeared after treatment. (C, D) Almost complete disappearance of skin lesions on the back after treatment.

A chest computer tomography (CT) scan detected a 4.1 cm × 2.5 cm pulmonary nodule in the middle lobe of the right lung, and revealed enlargement of the mediastinal, neck, and axillary lymph nodes (Figure 1 A1–D1). Physical examination revealed a large infiltrative erythema on the right side of the neck and shoulder, anterior chest wall, and back, with a long hard lump in the center and a slightly elevated skin temperature (Figure 2A and C). Multiple lymph nodes were detected in the neck and axilla with hard texture and poor mobility. Tumor marker tests revealed carcinoembryonic antigen (CEA) 8. 8 ng/ml, cytokeratin 19 fragment antigen 21 -1 (CYFRA 21-1) 35. 6 ng/ml, and cancer antigen 125 (CA-125) 101. 3 U/ml.

**Figure 3:**
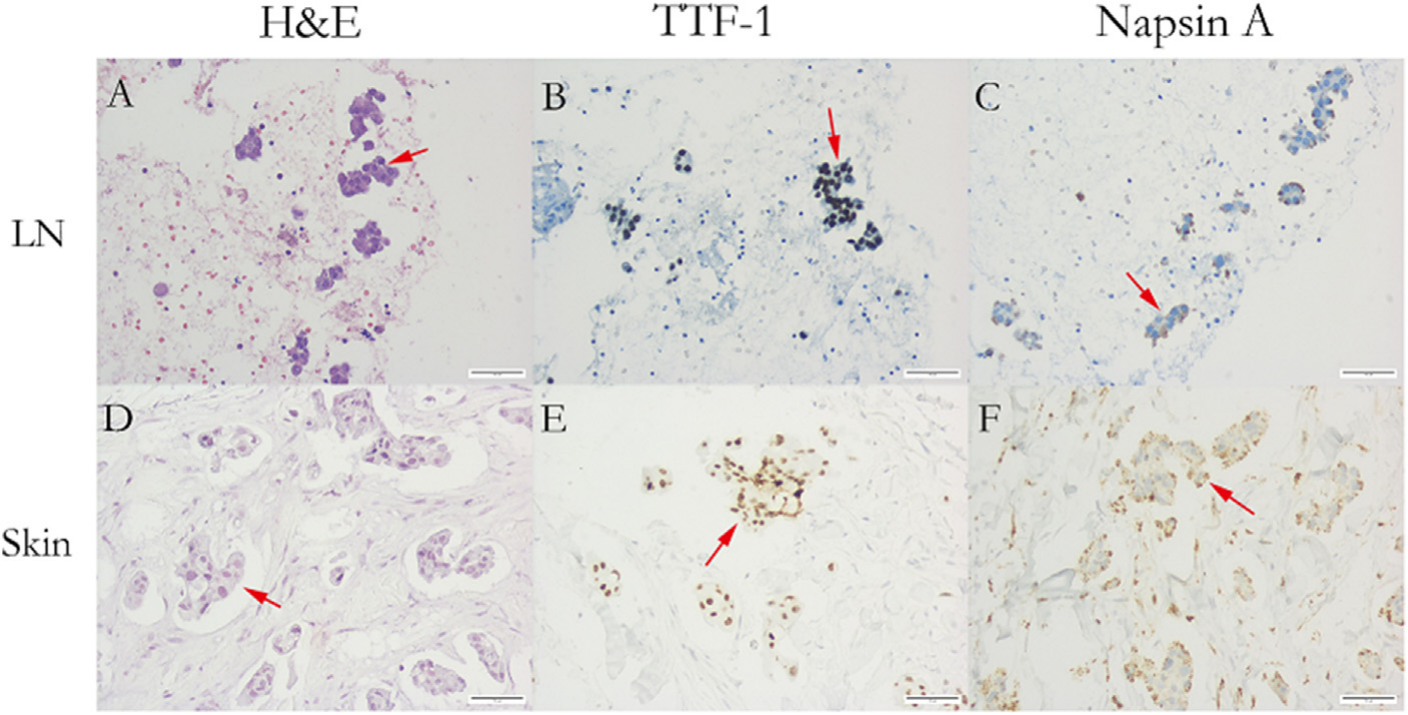
Adenocarcinoma confirmed by EBUS-TBNA and skin biopsy. (A) H&E staining of lymph nodes at hilum confirmed positive. (B) IHC using TTF-1 on lymph nodes at hilum confirmed focal positive.(C) IHC using Napsin A on lymph nodes at hilum confirmed positive.(D) H&E staining of skin biopsy sample confirmed positive.(E) IHC using TTF-1 on skin biopsy sample confirmed positive.(F) IHC using Napsin A on skin biopsy sample confirmed positive.

He underwent an endobronchial ultrasound-guided biopsy of the right hilar lymph nodes, and the lesion was diagnosed as adenocarcinoma. Immunohistochemical staining showed thyroid transcription factor 1 (TTF-1) (+), napsin A (+), cytokeratin 7 (CK7) (+), pan-cytokeratin (PCK) (+), p40 (−), and cytokeratin 20 (CK20) (−), confirming that the metastatic adenocarcinoma originated in the lung (Figure 3). The right shoulder skin biopsy also revealed adenocarcinoma. Immunohistochemical staining showed CK7 (+), CK20 (−), TTF-1 (+), napsin A (+), and special AT-rich sequence-binding protein 2 (SATB2) (−), supporting that the metastatic adenocarcinoma was of pulmonary origin (Figure 3). Enhanced CT scan of the abdomen, whole-body bone scan, and MRI of the head did not reveal other sites of tumor metastasis.

Next-generation sequencing (NGS) of the lymph node was performed by puncturing the tissue at the lung hilum. The results revealed an *EGFR* exon 21 L833V/H835L compound variant with 45.1% and 45.8% variant abundance, respectively, whereas *ALK, ROS-1, KRAS, BRAF, and HER2* were all negative. After careful review of the literature, we recommended afatinib or gefitinib for the patient. The patient chose the inexpensive gefitinib treatment (oral, 250 mg qd). The patient's skin symptoms almost completely disappeared after one month of treatment (Figure 2B and D). Moreover, the lung nodules and lymph nodes were significantly reduced and shrunken in the CT scan at the 3-month follow-up visit (Figure 1 A2–D2), indicating that the patient achieved a partial response (PR). At the time of submission (May 28th, 2022), the patient had been taking gefitinib orally for 18 months and no disease progression had occurred. The major treatment-associated adverse events were diarrhea (CTCAE grade 1) and scattered acne (CTCAE grade 1). The patient provided a written informed consent form and agreed to publish the details of the case.

## 3 Discussion

Our case showed potentially great efficacy of gefitinib in the treatment of NSCLC patients harboring L833V/H835L variants and accompanied by skin metastases. To our knowledge, our case has the longest PFS among the current reports of *EGFR*-TKI treatment in patients with L833V/H835L variants.

Compound EGFR variant is a combination of two or more independent variants in EGFR tyrosine kinase domain, with the common type being a combination of an EGFR classical variant and a rare variant. Simultaneous occurrence of two or more rare EGFR variants are extremely rare in lung cancer [3, 5]. The mechanism of action of *EGFR* TKI on compound variant is still unclear, and the prognosis of treatment of compound variant is controversial. Li et al. used molecular dynamics simulations to thoroughly investigate the molecular basis of gefitinib sensitivity of EGFR compound variants and single rare variants. The results showed that EGFR compound variants were more sensitive to gefitinib than single rare variants [6]. In terms of clinical treatment data, some studies found that patients with compound variants had an ORR of 6.2%–16.7% in first-line EGFR-TKI therapy, much lower than patients with single or classical EGFR variants (62.1%–84.6%), leading to varying degrees of shortened PFS and overall survival (OS) [7, 8]. However, as more compound variants are detected, data on EGFR-TKI treatment in this population are increasingly available. Kate et al. found that among all uncommon variants, patients with compound uncommon variants treated with oral EGFR TKI performed better, with mPFS of 9.1 months and mOS of 22.6 months, respectively [9]. A single-center retrospective study of EGFR variants in lung cancer patients showed that among patients receiving first- or second-generation EGFR-TKI as first-line therapy, patients with two uncommon EGFR variants had significantly better OS than those with a single uncommon variant [10].

EGFR L833V/H835L variant is a rare compound variant, with only a few case reports available. To our knowledge, only 12 cases carrying the H833V/H835L compound variant have been reported [1, 5, 11, 12, 13, 14, 15, 16, 17]. Moreover, fewer clinical references have detailed the clinical treatment information of this variant. By organizing these cases (Table 1), we found that the L833V/H835L compound variant seems to be more likely to occur in Asian males, and no correlation between the L833V/H835L compound variant and smoking status has been observed, and all of the above cases, except for E709K/L833V/H835L and R670W/H835L/L833V were simultaneous variants of L833V, H835L, and another rare gene; all of them were compound variants of only L833V and H835L, which seems to imply that the L833V/H835L compound variant is the main pattern of the presence of L833V and H835L, in NSCLC patients. All three generations of EGFR TKI-targeted drugs have been used for treatment. Based on the information on previous treatment and efficacy in these cases (Table 1), we recommended afatinib as a priority and the first generation TKI as a second choice, but the patient finally chose gefitinib for financial reasons. However, to our surprise, the patient's skin metastasis symptoms improved rapidly and the patient also achieved a very good PR result.

**Table 1:** Clinical characteristics and response to TKI drugs in 9 patients with NSCLC harboring L833V/H835L mutation.

Report year	Author	Sex	Age (year)	Pathological type	Smoking status	Mutations	EGFR-TKIs	PFS (months)
2004	Huang.	F	76	AD	NA	L833V/H835L	NA	NA
2006	RS Lai	M	NA	AD	NA	L833V/H835L	NA	NA
2011	TY Yang	M	89	AD	current smoker (light smoker)	L833V/H835L	gefitinib (second-line)	>8+
2012	Zhuang	M	48	AD	never smoked	L833V/H835L	NA	NA
2016	S. Frega	M	70	AD	past smoker	E709K/L833V/H835L	afatinib (first-line)	>2+
2018	BD Qin	M	36	AD	never smoked	R670W/H835L/L833V	gefitinib (first-line), afatinib (third-line)	>7
2018	LM Cao	F	77	AD	never smoked	L833V/H835L	gefitinib (first-line)	15
2020	X Long	M	65	AD	never smoked	L833V/H835L	afatinib (first-line)	>10
2021	T Li	M	75	AD	never smoked	L833V/H835L	afatinib (first-line), osimertinib (second-line)	12 (first-line), >1.5 (second-line)

Abbreviations: AD, adenocarcinoma; F, female; M, male; NA, not available.

Our patient's PFS was 18 + months at last follow-up, one of the best reported outcomes to date for patients with L833V/H835L variants treated with EGFR TKI. This may be partly related to the abundance of *EGFR* variant, which is a predictor of prognosis in advanced NSCLC [18]. In a retrospective study that included 194 patients having NSCLC with *EGFR* variants, Wang et al. found that median PFS was significantly greater in the high variant abundance group than in the low variant abundance group (12.7 vs. 8.7 months). The median PFS increased with an increase in *EGFR* variant abundance. They found that 26.7% was the best cut-off value for separating low and high *EGFR* variant abundance [19]. In our case, the variant abundance of *EGFR* exon 21 L833V and H835L reached 45.1% and 45.8%, respectively. The patient belongs to the high variant abundance group with a better prognosis, as indicated in this literature. However, Wang et al. only studied specific variant loci (located in exons 19 and 21) and did not list detailed variant types. Therefore, whether this conclusion applies to rare variants in the same exon needs to be further investigated. In addition, as we mentioned previously, there are conflicting opinions regarding the prognosis of patients with compound variants treated with EGFR TKI. More clinical reports are needed to enrich our data on the prognosis of rare compound variants in follow-up.

Another interesting point is the combination of cutaneous metastases in this patient. Patients with lung cancer usually initially present with respiratory symptoms, such as cough, sputum, dyspnea, and hemoptysis, or bone pain and headache (symptoms of metastatic lesions). Skin metastases are extremely rare as the first sign of internal malignancy and only occur in 0.8% of cases [20]. Skin metastases usually involve the anterior chest, abdomen, head, neck, and back, and often present as a single or multiple nodules, while papules, plaques, ulcers, and herpes zoster are less common [21]. The metastatic site of our patient was consistent with the literature, but the skin lesions presented as infiltrative flaky erythema, a rare manifestation that easily led to the misdiagnosis as a simple skin disease. In addition, cutaneous metastasis from visceral cancer often indicates a poor prognosis and is usually a sign of extensive metastatic malignancy [22]. In our case, the patient had cutaneous symptoms as the first symptom, and through follow-up, we found that cutaneous metastases were also the only distant metastasis in this patient. Interestingly, this patient had a rapid remission of skin symptoms after treatment with gefitinib. Subsequently, we reviewed the relevant literature and found that the concentration of gefitinib in tumor tissues was much higher than the plasma concentration in both animal models and human trials: 12-fold and 42-fold, respectively. The concentrations of gefitinib in skin and tumor tissues were similar in animal models [23]. However, the incidence of skin toxicity was lowest with gefitinib and similar with erlotinib and afatinib, and the frequency of grade 3 or higher skin rash was lowest with gefitinib and highest with afatinib [24]. These data also suggest that gefitinib may be a better choice in patients with cutaneous metastases, but more clinical evidence is still needed to verify this speculation.

## 4 Conclusion

Although there is now more evidence for the efficacy of afatinib in treating rare variants, there are very limited data on the treatment of L833V/H835L. Our case study and literature review suggest that NSCLC patients harboring the L833V/H835L variant are likely to benefit from gefitinib, especially for patients with cutaneous metastases. We may consider the actual situation of patients when making treatment choices in the future, so as to develop a more suitable individualized regimen for patients. Since the number of cases is too small and the data related to the L833V/H835L compound variant are almost entirely from case reports, the establishment of a database of rare compound variants seems necessary.

## Declarations

### Author contribution statement

All authors listed have significantly contributed to the investigation, development and writing of this article.

### Funding statement

This work was supported by 1.3.5 Project for Disciplines of Excellence, West China Hospital (ZYJC21042), Sichuan University for Qing Zhu.

### Data availability statement

Data included in article/supp. material/referenced in article.

### Declaration of interest’s statement

The authors declare no competing interests.

### Additional information

No additional information is available for this paper.
